# Detection of Zebrafish Retinal Proteins by Infrared Western Blotting

**DOI:** 10.21769/BioProtoc.4618

**Published:** 2023-02-20

**Authors:** Jingjing Zang, Stephan C. F. Neuhauss

**Affiliations:** Department of Molecular Life Science, University of Zurich, Zurich, Switzerland

**Keywords:** Infrared fluorescence western blot, Visual transduction, Circadian rhythms, Zebrafish, Retina

## Abstract

The zebrafish retina is a canonical vertebrate retina. Since the past few years, with the continually growing genetic toolbox and imaging techniques, zebrafish plays a crucial role in retinal research. This protocol describes a method to quantitatively evaluate the expression of Arrestin3a (Arr3a) and G-protein receptor kinase7a (Grk7a) in the adult zebrafish retina at protein levels by infrared fluorescence western blot. Our protocol can be easily adapted to measure protein levels in additional zebrafish tissues.

## Background

Circadian rhythms are the inner clocks that drive the daily fluctuations of physiology and behavior (Vatine et al., 2011; Brown et al., 2019; Frøland Steindal and Whitmore, 2019). The molecular mechanisms of circadian rhythms are based on a transcription-translation feedback loop, in which core clock genes’ mRNA and protein levels fluctuate in an approximate 24-h period. Certain core clock genes regulate the transcription of downstream clock-controlled genes, which in turn generate the circadian output. Strikingly, nearly 20% of the genes in zebrafish genome are thought to be regulated by the circadian clock (Li et al., 2013). Circadian fluctuations are commonly studied at the transcript level, using qRT-PCR or RNA-Seq. Circadian rhythms at the protein level are less accessible to study, partially because the daily changes are not very large. However, in numerous instances, circadian fluctuations in mRNA expression are not reflected in corresponding changes of protein levels. It is easy to conceptualize that proteins with a slow turnover rate will have stable overall protein levels even in the face of rhythmic mRNA expression. Hence, it is important to measure changes in protein concentration of any process, before interpreting their involvement in any observed rhythmic behavior.

In a recent study, we linked circadian expression of mRNA and proteins of the cone visual transduction cascade to visual sensitivity assayed by electrophysiological and behavioral means (Zang et al., 2021). The transcription of regulators of the visual transduction cascade, such as Recoverins, Arrestins, Opsin kinases, and Regulator of G-protein signaling displayed a robust circadian rhythm. Importantly, this rhythm was also observed at the protein level, with a relative delay likely due to translation. These changes in protein concentration can be directly correlated with changes in photoresponse kinetics. Electroretinography demonstrates that photoresponse recovery in zebrafish is delayed in the evening and accelerated in the morning. This rhythm can be reversed by an inverted light cycle, persists in constant darkness, and is disturbed by constant light, as is expected for a bona fide circadian rhythm. Interestingly, the cyclic expression of orthologous genes in the nocturnal mouse retina are anti-phasic to the ones of the diurnal zebrafish.

While general chemiluminescence western blotting can detect the analyzed proteins, infrared fluorescence western blot can simultaneously stain the protein of interest as well as the loading control. Additionally, the signal detection for infrared fluorescence western blot is straightforward.

Here, we describe the method of infrared fluorescence western blot to quantify the relative protein level change in adult zebrafish retina throughout the day, based on the standard polyacrylamide gel electrophoresis and protein transfer protocols provided by Bio-Rad and detection protocol provided by LI-COR Biosciences with modifications. The current protocol can be easily adapted to work with variable zebrafish tissues at different development stages or used to study protein level changes under other conditions, for example in the mutants or fish under drug treatment.

## Materials and Reagents

1. Nitrocellulose membrane, 0.45 µm (Bio-Rad, catalog number: 1620116)
2. Sterile filter, 0.45 µm (SARSTEDT AG&Co. KG, catalog number: 83.1826)
3. 4%–15% Mini-protean^®^ TGX^TM^ gels, 15-well comb, 15 µL (Bio-Rad, catalog number: 4561086)
4. Adult zebrafish (WIK wild-type strain)
5. Rabbit anti-ARR3a antibody (Renninger et al., 2011)
6. Rabbit antt-GRK7a antibody (Rinner et al., 2005)
7. Mouse anti-β-Actin (Sigma-Aldrich, catalog number: A1978)
8. Bovine serum albumin (BSA) (Roche, catalog number: 10735094001)
9. Tween^®^ 20 (Sigma-Aldrich, catalog number: P1379)
10. Ethylenediaminetetraacetic acid (EDTA) (Sigma-Aldrich, catalog number: EDS)
11. Sodium chloride (NaCl) (Sigma-Aldrich, catalog number: 71380)
12. TRIS (Biosolve, catalog number: 200923)
13. Triton^®^ X-100 (Fluka, catalog number: 93418)
14. Sodium deoxycholate (Sigma-Aldrich, catalog number: D6750)
15. Sodium dodecyl sulfate (SDS) (Roche, catalog number: 11667289001)
16. Protease inhibitor cocktail (PIC) tablets (Roche, catalog number: 04906845001)
17. Glycerol (Sigma-Aldrich, catalog number: G7757)
18. Bromophenol blue indicator (Fluka, catalog number: 32712)
19. β-mercaptoethanol (Sigma-Aldrich, catalog number: 444203)
20. Glycine (Sigma-Aldrich, catalog number: 50046)
21. Precision Plus protein kaleidoscope marker (Bio-Rad, catalog number: 1610375)
22. Phosphate buffer saline (PBS) (VWR, catalog number: 75801-006)
23. IRDye^®^ 800CW goat anti-rabbit IgG (LI-COR, catalog number: 925-32211)
24. IRDye^®^ 680RD goat anti-mouse IgG (LI-COR, catalog number: 925-68070)
25. Pierce^TM^ BCA Protein Assay kit (Thermo Scientific, catalog number: 23227)
26. Safe-Lock micro test tubes, 1.5 mL (Eppendorf, catalog number: EP0030120167)
27. Methylalkohol (MeOH) (Sigma-Aldrich, catalog number :1424109)
28. Hydrogen chloride (HCl) (Sigma-Aldrich, catalog number: 1099730001)
29. Sodium hydroxide (NaOH) (Sigma-Aldrich, catalog number: S5881)
30. Radioimmunoprecipitation assay (RIPA) buffer (see Recipes)
31. PIC (see Recipes)
32. 5× Laemmli sample buffer (see Recipes)
33. 10× running buffer (see Recipes)
34. 10× transfer buffer (see Recipes)
35. Phosphate buffered saline with Tween 20 (PBST) (see Recipes)
36. Blocking buffer (see Recipes)

## Equipment

1. Eyeball scoop (Chiru-Instrumente)
2. Micro scissors
3. Shaker
4. Sonicator (Bandelin Sonopuls)
5. PowerPac basic power supply (Bio-Rad, catalog number: 164-5050)
6. Mini-PROTEAN^®^ Tetra vertical electrophoresis cell (Bio-Rad, catalog number: 165-8004)
7. Mini Trans-Blot^®^ module (Bio-Rad, catalog number: 170-3935)
8. Odyssey^®^ DLx imaging system (LI-COR)
9. NanoDrop^TM ^One (Thermo Scientific, catalog number: ND-ONE-W)
10. pH meter (SCHOTT Instruments, model: Lab 850)

## Software

1. Image Studio software (LI-COR, version: 5.2, https://www.licor.com/bio/image-studio-lite/)
2. Microsoft 365 Excel

## Procedure

**Figure 1.**
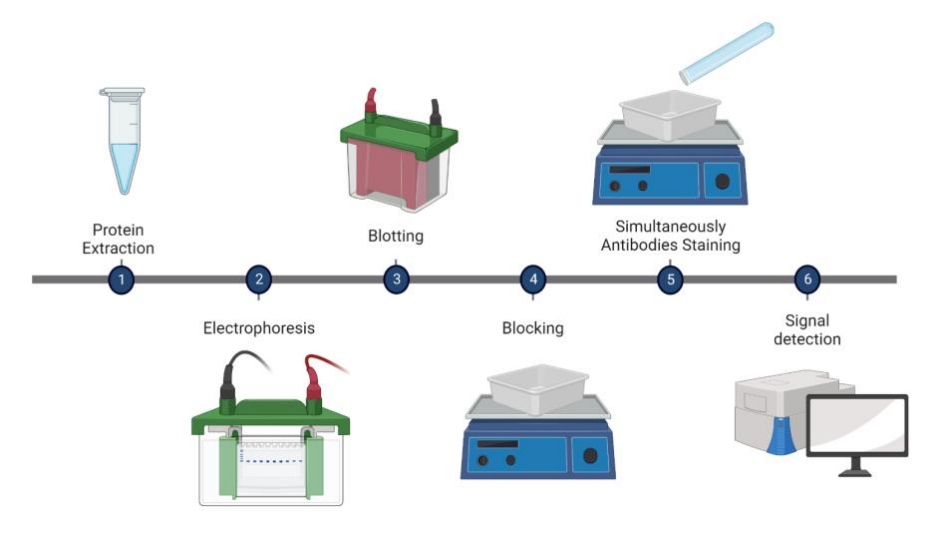
Western blotting workflow

Pictorial methodology is illustrated in Figure 1.

- **Eye dissection**
  1. Place the fish in ice water at 1–2 °C at the desired time of the day.
  2. Leave the fish in the ice water for 2–5 min and decapitate the fish.
  3. Remove the eyeballs with a scoop (Figure 2B) and cut the white optic nerve using micro scissors (Video 1).
    **Figure 2.**
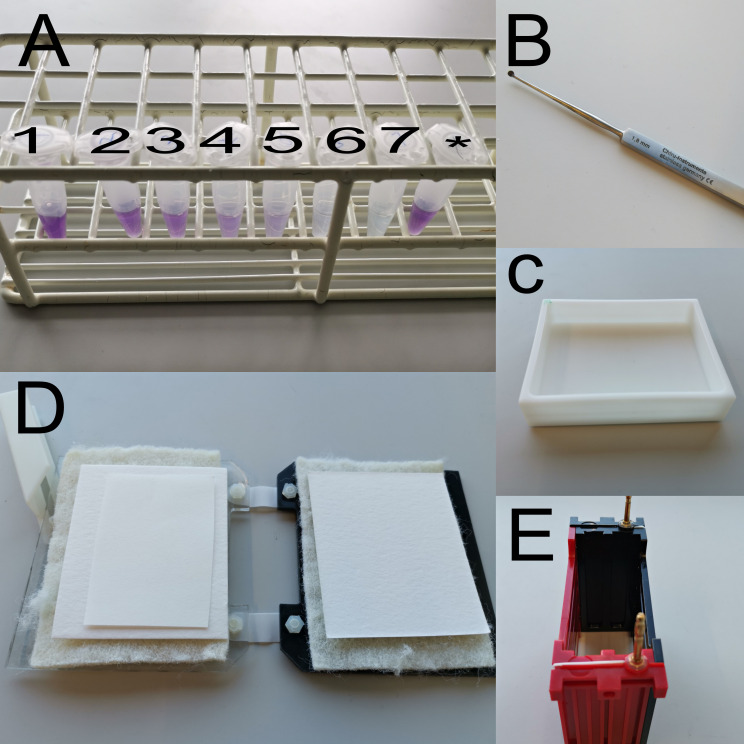
Items used during western blotting. (A) BSA standards (1–7) and protein sample (*). (B) Eye scoop. (C) Incubator. (D) Transfer cassette. (E) Transfer module.
    **Video 1.**
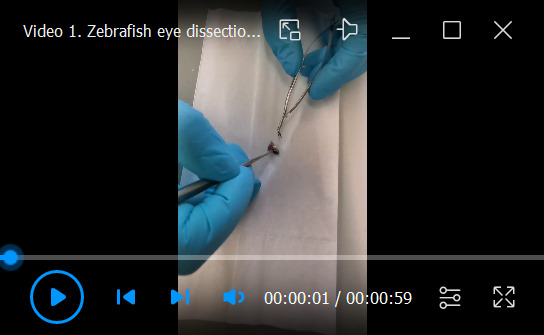
Zebrafish eye dissection
  4. Shock-freeze the eyes (3–4 eyes per sample) in liquid nitrogen.
*Notes:*
    - *In the original paper, western blot was used to evaluate protein level changes in a 24-h period. The eyes were collected every 3 h at eight different time points. Therefore, it was easier to store the eyes and perform the protein extraction for all the samples at once. Depending on the purpose of the experiment, the eyeballs can be collected directly in RIPA buffer and processed for the protein extraction right after the dissection.*
    - *The dissection should be carried out under dim red light if light adaptive changes are to be avoided.*
- **Protein extraction**
  1. Perform all steps on ice.
  2. Collect eyes and put them in 150 µL of cold RIPA buffer with 1× PIC, which is added right before the extraction.
  3. Homogenize eyes with sonicator three times for 3 s at 70% maximum power.
  4. Incubate on shaker at 4 °C for 2 h, centrifuge at 5,590 *× g* for 30 min at 4 °C, and transfer the supernatant to a fresh 1.5 mL tube.
  5. Perform BCA assay to determine the protein concentration:
    - Use 1 ampule (1 mL, 2.0 mg/mL) of albumin standard from the Pierce^TM^ BCA Protein Assay kit to prepare BSA dilutions in RIPA buffer according to Table 1.
*Note: Duplicates are normally prepared.*
    - Prepare the BCA working reagents (WR) and calculate the total amount of WR according to sample to WR ratio (1:20). Prepare WR by mixing reagent A from the kit with reagent B (50:1).
    - Mix well 15 µL of each standard with 300 µL of WR in separate 1.5 mL tubes. Mix well protein lysate with 300 µL of WR in another 1.5 mL tube. Incubate the tubes in a heating block at 37 °C for 30 min. Cool them down to room temperature (Figure 2A).
    - Use a photospectrometer to determine concentration (we used a NanoDrop device).
      i. Input the number of replicates and concentrations of BSA standards according to Table 1.
      ii. Measure the protein concentration for each standard and replicate according to NanoDrop instructions.
      iii. Measure the sample concentration according to the instructions. The result will be shown on the screen and can be exported. Continue with the following steps or store the lysate at -80 °C until use.
      **Table 1.** Dilutions for preparing BSA standards

Vial	Volume of RIPA Buffer(µL)	Volume of Source of BSA (µL)	Final BSA Concentration (µg/ml)
1	0	40 of Stock	2000
2	10	30 of Stock	1500
3	20	20 of Stock	1000
4	20	20 from vial 2 dilution	750
5	20	20 from vial 3 dilution	500
6	30	10 from vial 5 dilution	125
7	40	0	0
- **Electrophoresis**
  1. Mix lysates with 1× loading buffer containing 5% β-mercaptoethanol freshly added and place the samples on ice.
  2. Insert gel(s) into the electrophoresis cell, fill with 1× running buffer completely covering the gel, remove combs, and check/remove bubbles in each well.
  3. Load 10 µL of Precision Plus protein kaleidoscope marker and 20 µg of each sample.
*Note: An asymmetric loading scheme will make it easier to recognize the orientation of the membrane later.*
  4. Run electrophoresis at around 100 V until the blue front band reaches the bottom of the separating gel.
*Note: The higher the voltage is applied, the faster the proteins travel. However, the bands sometimes do not run at the same height throughout the gel (“smile effect”).*
  5. Remove the gel from electrophoresis cell.
- **Blotting**
  1. Work under the fume hood with good ventilation.
  2. Prepare the electroblotting system:
    - For each gel, cut one piece of nitrocellulose membrane and two pieces of filter paper to the size of the gel.
    - Equilibrate the membrane, filter paper, and two filter pads in 1× transfer buffer containing 20% MeOH in an incubator (Figure 2C).
  3. Mount the transfer cassette according to the scheme and remove air bubbles by gently pressing on the gel or membrane (Figure 2D).
    - Cathode pole (black)
    - Filter pad
    - Filter paper
    - Gel
    - Membrane
    - Filter paper
    - Filter pad
    - Anode pole (white)
  4. Insert the transfer cassette into the Trans-Blot^®^ module (Figure 2E), insert an ice block and a magnetic stir bar, and fill with 1× transfer buffer containing 20% MeOH completely covering the transfer module. Place the whole transfer module on a magnetic stirrer plate, which allows continuously stirring the buffer inside the module to keep the temperature low.
  5. Blot at 100 V/350 mA for around 40 min for protein Arrestin3a (Arr3a) and around 1 h for G-protein receptor kinase7a (Grk7a).
*Note: Blotting time varies according to protein size. In this protocol, we focused on Arr3a (around 41 kDa), Grk7a (around 63 kDa), and loading control β-Actin (around 42 kDa). In general, for proteins smaller than 50 kDa, blotting takes around 40 min. For proteins between 50 and 150 kDa, blotting takes around 1 h. For proteins larger than 150 kDa, blotting takes around 1 h in 1× transfer buffer without MeOH.*
  6. Transfer the membrane into a dish with PBS and mark membrane on one of the corners to remember which side is the top side.
- **Antibody staining**
  1. Place the membrane in a container (Figure 2C) and rinse twice for 5 min with 1× PBST.
  2. Block for 1 h in blocking solution on the shaker and shake gently.
  3. Prepare the first antibodies (rabbit anti-Arr3a: 1:4,000 and mouse anti-β-Actin: 1:6,000, or rabbit anti-Grk7a: 1:3,000 and mouse anti-β-Actin: 1:6,000) in blocking solution at 4 °C and incubate the membrane overnight at 4 °C on the shaker.
*Note: The first antibody solution can be kept at 4 °C and reused later. Depending on the antibody, this solution may be reused five times.*
  4. Wash for 5 min in blocking solution at room temperature.
  5. Wash three times for 10 min in 1× PBST.
  6. Prepare the secondary antibodies (IRDye^®^ 800CW goat anti-rabbit IgG and IRDye^®^ 680RD goat anti-mouse IgG, both at 1:20,000) in blocking solution and incubate the membrane for 1 h on the shaker. Protect from light by covering the container completely with foil or placing it in a black box from now on.
  7. Wash three times for 5 min in 1× PBST.
- **Signal detection**
  1. Clean the scanning area of the imager with water and place the membrane.
  2. Define the area of interest in the Image Studio software and scan.
  3. Acquire images at 700 nm to visualize the signal of Arr3a or Grk7a, and at 800 nm to visualize the signal of β-Actin.
*Note: A membrane image example is shown in Figure 3 (Zang et al., 2021).*

## Data analysis

1. Perform the initial analysis with Image Studio.
2. Draw a rectangle around each band. The signals at 700 and/or 800 nm will be calculated automatically by the software.
3. Normalize the Arr3a or Grk7a signal to β-Actin signal in the appropriate software (e.g., Excel). At least three independent experiments should be performed.
*Note: The daily rhythms in the protein level of Arr3a and Grk7a were evaluated in the original paper. Statistical analysis was performed by “RAIN” as previously described (Thaben and Westermark, 2014).*
  **Figure 3.**
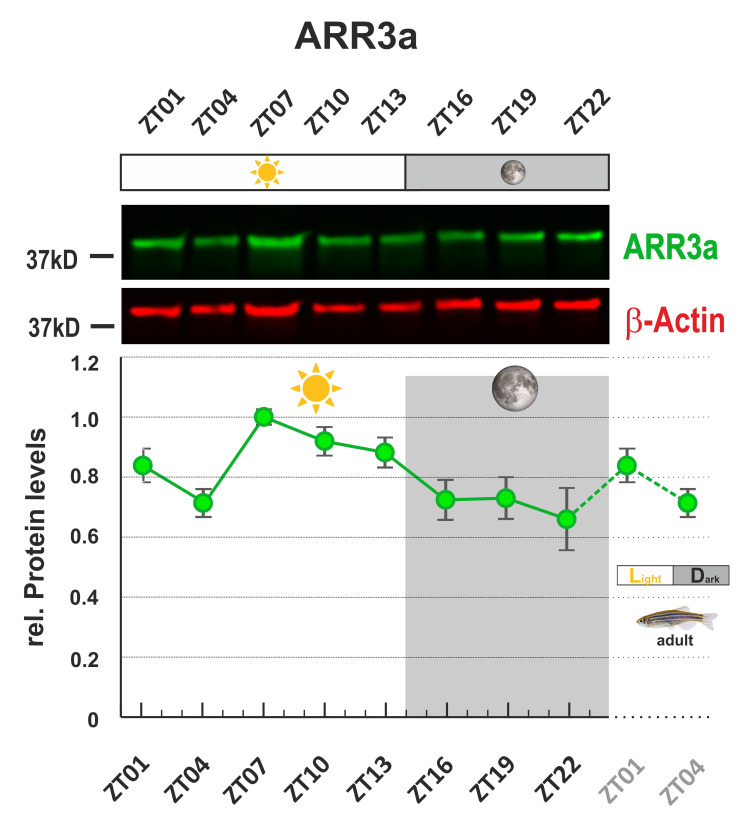
Arr3a protein levels show daily changes in adult zebrafish eyes. This figure was modified according to Figure 3 (Zang et al., 2021; https://elifesciences.org/articles/68903). One sample image of infrared fluorescence western blot was shown. Protein of interest was Arr3a. β-Actin was used as loading control. Eyes were collected every 3 h throughout a 24-h period. The highest protein level was normalized to 1. ZT = Zeitgeber time.

## Recipes

1. **RIPA buffer**
  NaCl, 150 mM
  Triton X-100, 1% v/v
  Sodium deoxycholate, 0.5% w/v
  Tris, 50 mM, pH 8 adjusted with HCl and/or NaOH
  EDTA, 1 mM
  SDS, 0.1% w/v
  Filter-sterilize
2. **PIC**
  Dissolve one PIC tablet in 1.5 mL of double-distilled H_2_O to obtain 7× stock solution
  Add 1× PIC to RIPA buffer before preparing lysates
3. **5× Laemmli sample buffer**
  Tris, 250 mM, pH 6.8 adjusted with HCl and/or NaOH
  Glycerol, 50% v/v
  SDS, 10% w/v
  Bromophenol blue, 1% w/v
  β-mercaptoethanol, 5% v/v (add right before use)
  Filter-sterilize
4. **10× running buffer**
  Tris, 250 mM
  Glycine, 1.9 M
  SDS, 0.50% w/v
  Autoclave
5. **10× transfer buffer**
  Tris, 200 mM
  Glycine, 1.5 M
  Autoclave
6. **PBST**
  1× PBS pH 7
  Tween 20, 0.1% v/v
7. **Blocking buffer**
  1× PBST
  BSA, 1% w/v
